# Wheat quality: A review on chemical composition, nutritional attributes, grain anatomy, types, classification, and function of seed storage proteins in bread making quality

**DOI:** 10.3389/fnut.2023.1053196

**Published:** 2023-02-24

**Authors:** Anam Khalid, Amjad Hameed, Muhammad Farrukh Tahir

**Affiliations:** ^1^Department of Biochemistry, University of Jhang, Jhang, Pakistan; ^2^Nuclear Institute for Agriculture and Biology (NIAB), Faisalabad, Pakistan

**Keywords:** wheat, HMW-GS, LMW-GS, grain anatomy, nutritional quality

## Abstract

Wheat (*Triticum aestivum* L.) belonging to one of the most diverse and substantial families, Poaceae, is the principal cereal crop for the majority of the world’s population. This cereal is polyploidy in nature and domestically grown worldwide. Wheat is the source of approximately half of the food calories consumed worldwide and is rich in proteins (gluten), minerals (Cu, Mg, Zn, P, and Fe), vitamins (B-group and E), riboflavin, niacin, thiamine, and dietary fiber. Wheat seed-storage proteins represent an important source of food and energy and play a major role in the determination of bread-making quality. The two groups of wheat grain proteins, i.e., gliadins and glutenins, have been widely studied using SDS-PAGE and other techniques. Sustainable production with little input of chemicals along with high nutritional quality for its precise ultimate uses in the human diet are major focus areas for wheat improvement. An expansion in the hereditary base of wheat varieties must be considered in the wheat breeding program. It may be accomplished in several ways, such as the use of plant genetic resources, comprising wild relatives and landraces, germplasm-assisted breeding through advanced genomic tools, and the application of modern methods, such as genome editing. In this review, we critically focus on phytochemical composition, reproduction growth, types, quality, seed storage protein, and recent challenges in wheat breeding and discuss possible ways forward to combat those issues.

## 1. Overview

Wheat is the most extensively cultivated cereal grain around the globe and holds a crucial place in agriculture (1–4). It is a principal nutriment for 36% of the world’s populace and is propagated in 70% of the world’s cultivated regions. (5, 6). Internationally, wheat supplies approximately 55% of the carbohydrates and 21% of food calories consumed worldwide (6–8). It beats every other single grain crop (including rice, maize, etc.) in production and acreage and is grown across a broad range of climatic situations (9); it is therefore the most significant grain crop on the entire planet (Table 1).

**Table 1:** Classification of *Triticum aestivum* (10).

Kingdom	Plantae (Plants)
Sub-kingdom	Tracheobionta (Vascular plants)
Super division	Spermatophyta (Seed plants)
Division	Magnoliophyta (Flowering plants)
Class	Liliopsida (Monocotyledon)
Subclass	Commelinidae
Order	Cyperales
Family	Poaceae/Gramineae (grass family)
Genus	Triticum (wheat)
Species	*Triticum aestivum* (common wheat)

Wheat is of supreme importance among cereals mainly because of its grains, which comprise protein with exclusive physical and chemical attributes. It also encompasses other useful components, such as minerals (Cu, Mg, Zn, Fe, and P), protein, and vitamins (riboflavin, thiamine, niacin, and alpha-tocopherol), and is also a valuable source of carbohydrates (11). However, wheat proteins have been found to lack vital amino acids; for example, lysine and threonine (12–14).

Wheat production and quality could possibly be enhanced through the development of new and improved varieties that are able to produce a superior yield and perform better under various agro-climatic stresses and conditions (15). It is the common consensus that the diversity of germplasm in breeding material is an essential component in plant breeding (16, 17).

### 1.1. Wheat background

**Figure 1:**
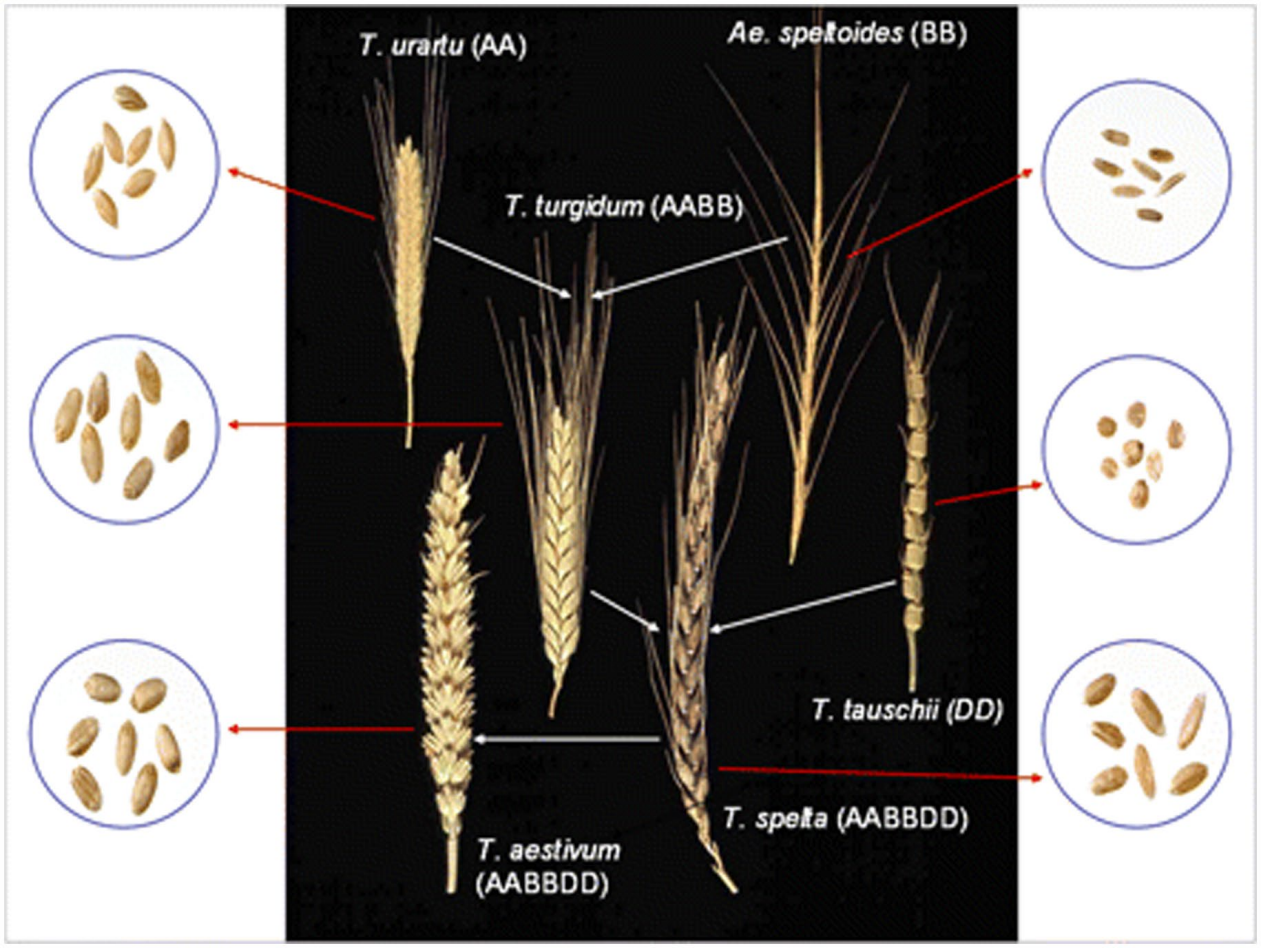
The evolutionary and genome relationships between cultivated bread and durum wheat and related wild diploid grasses, showing examples of spikes and grain.

Wheat was first cultivated approximately ten thousand years ago during the ‘Neolithic Revolution’, which saw a shift from hunting and collecting food to stable land management. Diploid, i.e., genome AA, einkorn, and tetraploid, i.e., genome AABB, emmer, were the first types of wheat to be grown and, according to their hereditary relationship, they originated in the southeastern regions of Turkey (18, 19). Cultivation expanded to the Nearby East almost nine thousand years ago with the first appearance of hexaploid wheat (20, 21). The evolutionary and genome relationships between cultivated bread and durum wheat and related wild diploid grasses, showing examples of spikes and grain, are shown in Figure 1 (20).

The previously grown forms of wheat were essentially landraces from wild populations that were carefully chosen by farmers, probably because of their higher yields. However, domestication is also linked with the genetic trait selection of wheat, which is detached from that of its wild ancestors. Two characteristics are of significant importance, the first being spike-shattering loss at maturity, which causes a loss of seeds at the time of harvest (22). It is a vital characteristic for certifying the dissemination of seeds in genuine populations. The second is the non-shattering characteristic, which has been deduced through alterations at the brittle rachis (Br) locus (23) and the conversion from husked to free-threshing nude forms, in which the outer sterile husk attaches firmly to the seeds. The unrestricted configurations merge from a deviant on the Q locus that alters the influence of receding mutations at the pertinacious grain husk (Tg) locus (18, 24, 25).

**Figure 2:**
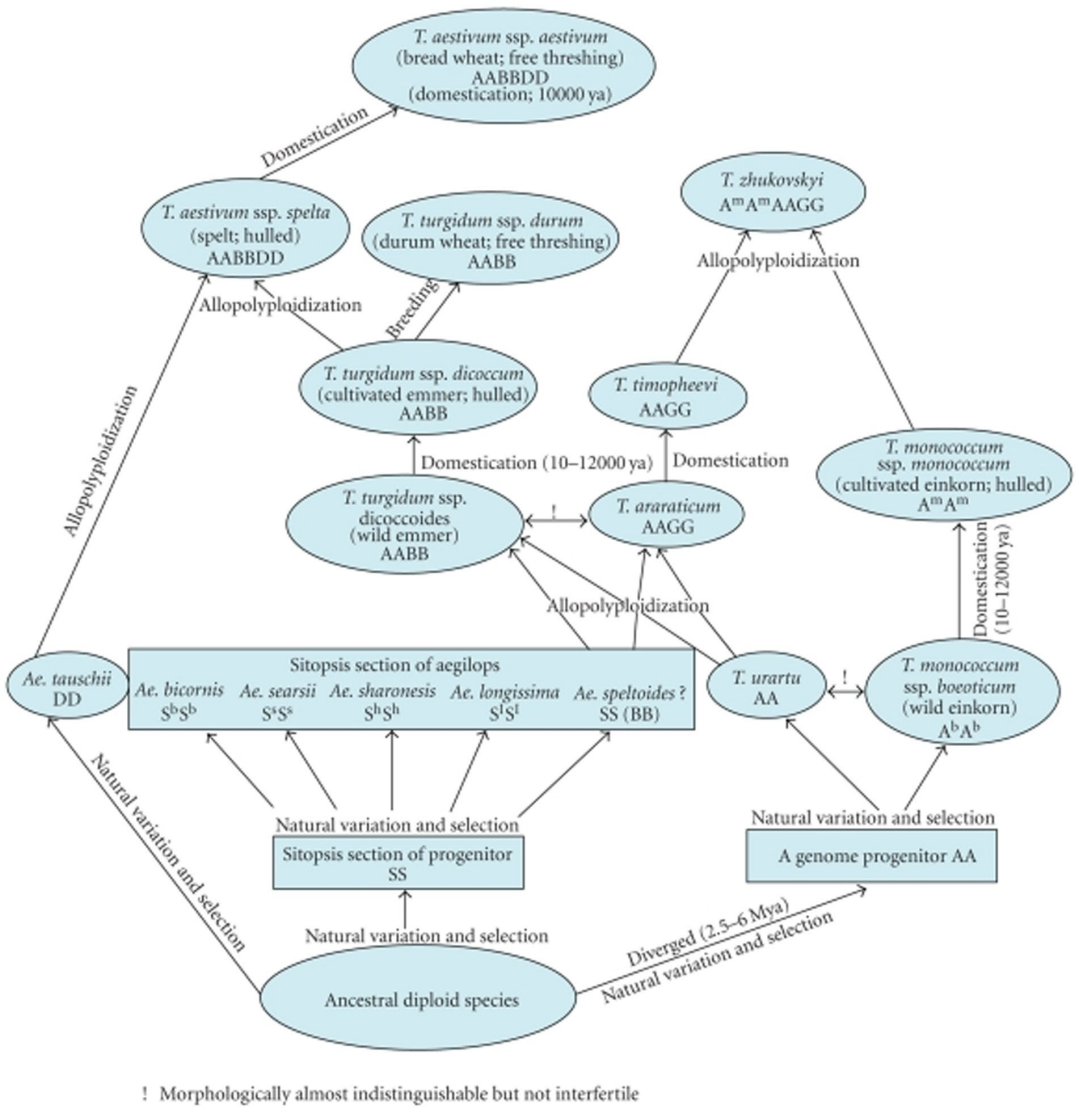
Evolutionary history of wheat.

The haploid content of DNA regarding wheat’s six sets of chromosomes (*Triticum aestivum* L. em Thell, 2n = 42, AABBDD) is almost 1.7 × 1,010 base pair. It is approximately 100× greater than that of the genome of *Arabidopsis*, 40× that of rice, and nearly 6× that of maize (20, 26). The majority of the DNA sequence of bread wheat is derived from polyploidy, with substantial duplication, in which, repetitive DNA sequences make up 80% of the entire genome (27, 28). The typical wheat chromosome is approximately 810 MB, 25× greater than the usual rice chromosome. The developmental history of wheat is illustrated in Figure 2 (29).

At present, approximately 95% of wheat cultivated throughout the world is hexaploid bread wheat, and the residual 5% is tetraploid durum wheat (21). The latter is better adapted to the arid Mediterranean environment than to bread wheat and is frequently referred to as pasta wheat to manifest its ultimate specific usage (30). Small quantities of other species of wheat, like emmer, spelt, and einkorn, are cultivated in few areas, including the Balkans, Spain, Turkey, and the Indian subcontinent (20, 31).

### 1.2. Taxonomic classification

See Table 1.

### 1.3. Types of wheat

The genus name for wheat, i.e., *Triticum*, is derived from the Latin word ‘tero’ (I thresh). The modern name, *Triticum aestivum*, represents hexaploid bread wheat with genomes A, B, and D, differentiating it from tetraploid macaroni wheat, which is *Triticum durum,* comprising genomes A and B, and is consumed predominantly for the production of pasta. Nowadays, bread wheat (*Triticum aestivum*) is the most extensively grown wheat. It is a hexaploid type of free-threshing wheat (genome AABBDD). According to Nesbitt and Samuel, it stemmed from the recent hybridization of the diploid (DD) Aegilops tauschii var. strangulate and an allotetraploid wheat (AABB) no longer than 8,000 years ago (32).

*Triticum aestivum* and *Triticum durum* consist of seven chromosome pairs (2n =14). Wheat has been cultivated in the form of spring or winter crops. In extremely cold areas, spring varieties of wheat have been propagated during spring so that they can grow and ripen rapidly and can be harvested before the arrival of the autumn snowfall. Within more temperate areas, winter wheat is propagated prior to the onset of the winter snowfall that otherwise covers the saplings, resulting in vernalization and allowing quick growth when the snow thaws in the spring. In warm environments, peculiarity in spring and winter wheat is almost futile. The point of significant difference is early or delayed maturity (33). Types of wheat have been frequently differentiated according to endosperm texture, seed coat, dough strength, color, and planting season. These are concisely explained as follows (34).

#### 1.3.1. White and red wheat

Red wheat variants usually have greater latency than white variants and have therefore been preferred in environments that are favorable to harvesting before germination. White wheat variants are suitable for growth in regions that are arid throughout the course of ripening and harvesting and are ideal for manufacturing flat noodles and bread (35).

#### 1.3.2. Soft and hard wheat

Although there are several wheat varieties grown around the world, they all fall into two essential categories (Table 2) with distinct properties: hard wheat and soft wheat (37). Variety and seed stability in hard and soft wheat are related to resistance to being crushed (35).

**Table 2:** Principal cultivars of soft and hard wheat cultivated in the world (36).

Hard wheat varieties	
Durum	Extremely hard, radiant, pale tinted grain used to form semolina flour in order to make pasta & bulghur, rich in gluten protein, high absorption of water
Hard red winter	Hard, brown in color, protein-rich, used for hard-baked products and bread as well as pastry flours to elevate protein intended for pie crusts
Hard red spring	Hard, brown in color, protein-rich, used for bread and hard-baked products. Usually used in bread and gluten-rich flours
Hard white	Hard, opaque, light in color, pale, medium protein content, planted in arid and temperate regions. Used for bread/brewing
Soft wheat varieties	
Soft red winter	Soft, low protein content, low water absorption, used for muffins, pie crusts, biscuits, and cakes.
Soft white	Soft, bran is deficient in pigment, low protein content, low water absorption, grown in moderately humid regions. Used for noodles, crackers, and wafers.

#### 1.3.3. Weak and strong wheat

The production of leavened bread is chiefly restricted to the full DNA sequence code for the proteins required for making a strong and elastic dough that is appropriate for capturing gas bubbles during fermentation, allowing the dough to upsurge. The exclusive pliable attributes of dough are principally the result of the amount and type of gluten present. Varieties with high gliadin glutenin contents are viscous and produce expansible doughs, which are appropriate for preparing cookies, for example, while varieties with a small gliadin glutenin content have greater strength and elasticity, which is ideal for bread making.

The difference in alleles in high-molecular-weight glutenins is nearly associated with the quality of bread making and the ability of the dough to withstand. Bread wheat varieties have three or five main high-molecular-weight glutenin subunits (38, 39). Glu-D1 genes encode two of these subunits, one or two are encoded by Glu-B1, and either none or one may be encoded by Glu-A1 (35, 40). More than 50% of the difference in the baking potential and viscoelastic attributes of dough depends on the wheat’s composition of high-molecular-weight subunits of glutenin (35).

#### 1.3.4. Spring and winter wheat

These varieties are diverse in their need for a frozen phase to allow normal development and reproductive growth. This need for vernalization has been vigorously affected by changes at the Vrn-1 position (present on the long arms of group 5 chromosomes), such as Vrn-B1, Vrn-A1, and Vrn-D1, and their superficial adjustment by negligible flower-inducing genes (41). Spring habits result from the dominant Vrn-la on any of the three genomes of wheat and the presence of the recessive, while winter habits result from alleles on Vrn-1b on all three genomes. However, Vrn-1 genes have a close association with the genes providing resistance to cold (42) and, thus, persist in winter (34).

Wheat development is largely determined by temperature, the requirement of a cold phase, variety, and plant responses to the corresponding lengths of dark and light periods during their developmental phase. As previously stated, winter wheat variety maturation was found to be accelerated due to the flowering process in plants, i.e., with low-temperature exposure, usually 3–10°C, for 6 to 8 weeks. Growth has also been enhanced through long day exposure which meaning growth is enhanced through longer period of light as the days lengthen in spring. As the varieties differ in their responses to vernalization, temperature, photoperiod, and the extent of interaction between certain factors, they vary continuously in their maturation rate and, therefore, contribute to the broader distribution and adaptation of wheat in agriculture globally (34, 43).

### 1.4. Vegetative growth

#### 1.4.1. Development of wheat seed

**Figure 3:**
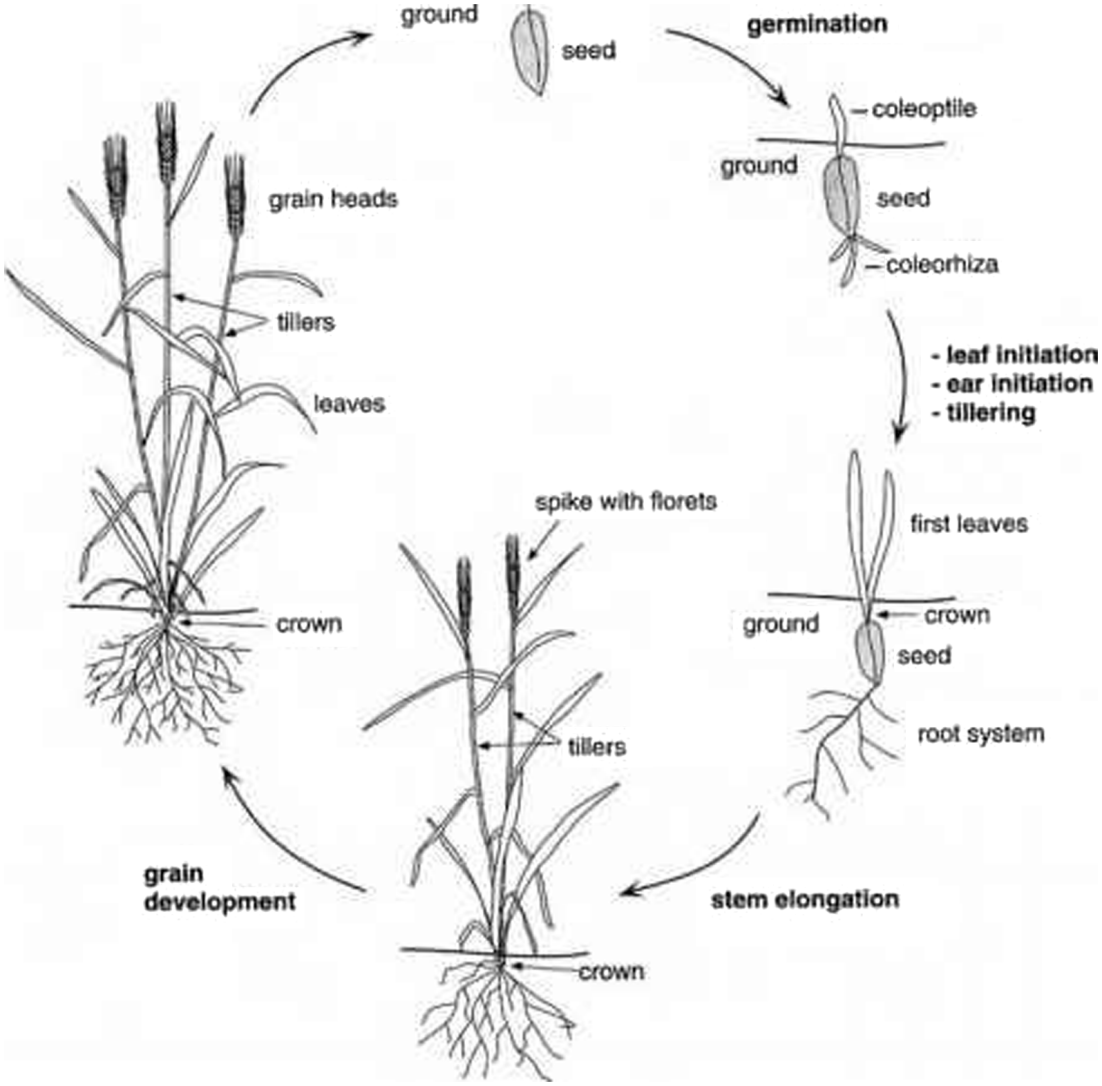
Life cycle of wheat.

Wheat seeds require moisture levels of 35–45% for germination (35, 44, 45). During propagation (Figure 3) (46), the adventitious side root outspreads earlier than the coleoptile. Seminal roots are generated in relation to the node of the coleoptile. When coleoptile arises from the soil, its development halts and the first true leaf propels to its end. Seedlings rely on nutrients and energy supplied through the endosperm until their first leaf is photo-synthetically efficient (35).

#### 1.4.2. Root growth

More than one node can grow in the soil based on sowing depth, all exhibiting roots (47). The root axis grows during expected periods in association with shoot growth, and the overall number of roots generated is linked with the number of leaves present on lateral branches and the extent of tillering (45). Roots emerging from lateral branches usually spread once the tillers have developed three leaves. A variety’s root development is analogous to its apex extension (35).

#### 1.4.3. Leaf growth

After germination, the apex of a vegetative shoot gives rise to secondary leaf primordia. Leaf primordial count can differ from seven to fifteen (35) and is influenced by light strength, the nutritional level of the plant, and temperature. Temperature imparts a significant effect on the emergence of leaves and expansion. The lowest temperature withstood for the expansion of leaves is approximately 0°C, the optimal temperature is 28°C, and the maximum is 38°C (35, 48).

#### 1.4.4. Stem development

Stem elongation overlaps with the growth of tillers, leaves, inflorescence, and roots (49). Stem elongation initiates when the maximum number of florets are present on the evolving spike, introducing the stamen’s initial identifiable stage, which resembles almost terminal spikelet development. The fourth internode, with nine leaves, is the first to extend in spring wheat, whereas the stem’s lower internodes remain short. Once an internode is extended partly to its ultimate extent, the internode above it starts to extend. This continues until the elongation of the stem is complete, generally close to anthesis.

The peduncle is the last segment to extend. The height of the wheat plant extends from 30 to 150 cm depending on the variety and the propagating state. Alterations in plant stature are generally attributed to the differences in internode dimensions and not to the internode number (35, 50).

#### 1.4.5. Tiller growth

The first lateral branches to arise are formed between the coleoptile axils and the first true leaf. Generally, three intervals between two successive leaves divide the leaf emergence and its subtended tiller. In winter wheat, small numbers of tillers develop in winter or autumn if circumstances are moderate. The main shoot and initially developed tillers fulfill their growth and develop granules in spring and winter wheat (51).

### 1.5. Reproduction

Wheat is principally an intra-floral pollinated crop. However, the rate of outcrossing is up to 10% or greater on the basis of genotype, population density, and environmental conditions. Cross-fertilization due to wind depends greatly on physical aspects such as excessive humidity and warm climates (52). Dry, warm climates give rise to increased cross-fertilization rates, i.e., 3.7–9.7% in comparison to the insignificant cross-fertilization rates of 0.1% under high-moisture conditions (53). Allogamy in wheat has been observed as high as 1–2%. Flowering time and duration depend on geographical location. Sunny climates and temperatures of at least 11–13°C are necessary for blooming (54).

#### 1.5.1. Spike growth prior to anthesis

The shift to propagative growth takes place close to apical cupola elongation, once the core shoot has almost three complete leaves. Floret division initiates in the crucial portion of the spike and continues both up and down as spikelet induction is completed. It creates a growth pyramid inside the prickle, which continues throughout grain growth and anthesis. Terminal spikelet instigation indicates the completion of spikelet formation (55).

During pre-anthesis, various developmental phases synchronize with one another (46). Kirby identified a difference of several weeks in the instigation of numerous shoots on a plant, which is decreased to just a few days in the period of spike appearance. Likewise, variation in the spikelet initiation period between the two early clusters in a fused flower could span 2 days; however, the difference in the duration of meiosis of these flowerets is around 6 h (56). Once the pollen-comprising stamen part elongates up to 1 mm and is green, meiosis takes place instantaneously in the pistil and anthers (55). The duration for which wheat flowers remain open varies from 8 to 60 min depending on environmental conditions and genotype (57).

#### 1.5.2. Kernel growth

The ratio of multiplication of the endosperm cell is affected by water stress, light intensity, genotype, and temperature (58, 59). The accumulation of starch starts at 1 to 2 weeks following anthesis and begins a 2 to 4-week period of direct rise in a dry mass of kernel (60, 61). The development and ultimate mass of a single kernel are determined by spikelet and floret site; grains that are developed in proximal florets and middle spikelets are generally very large (56, 60, 62). When rain coincides with harvesting, germination takes place. Seeds ripened in cold conditions are more latent compared to seeds matured in warm environments (63).

### 1.6. Grain anatomy

Wheat grain is divided into three main segments, all structurally and chemically distinguished from erstwhile. These are: the germ, also called the embryo, which is located at a single end of the grain in the form of a tiny, yellow mound, simply differentiated from the rest of the kernel; the endosperm, which covers a larger part of the whole grain and supplies nutrition to the developing plant as the kernel evolves; and the external seed crust and cover lying underneath, which contains protein cells that cover the whole kernel and protects the embryo and the endosperm on or after injury during the grain’s subsistence (latent phase) (64). Regarding the unique roles of all three parts, a significant difference exists in the chemical composition of their constituents and, therefore, a broader variation is found in their nutritional value (65).

**Figure 4:**
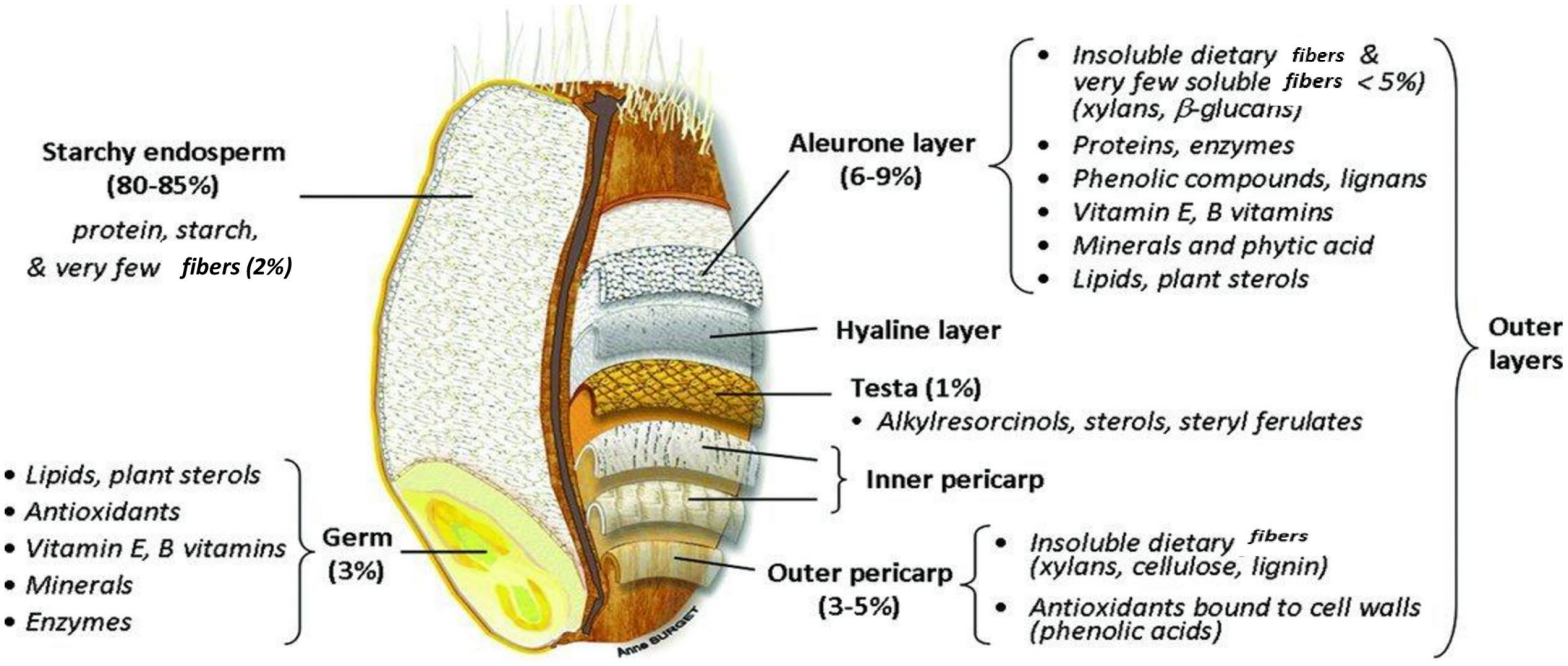
Chemical constituents in different parts of wheat grain.

Wheat kernels are usually elliptical, though different types of wheat have kernels that vary from virtually long, trampled, slender, and spherical in shape. The length and mass of the kernel are typically around 5–9 mm and 35–50 mg. It features a crinkle below the lateral side and it was therefore initially associated with the wheat flower. The wheat kernel (Figure 4) (65) encompasses 2–3% of the germ, 13–17% of the bran, and 80–85% of the mealy endosperm (entire elements altered to dehydrated material) (66).

**Figure 5:**
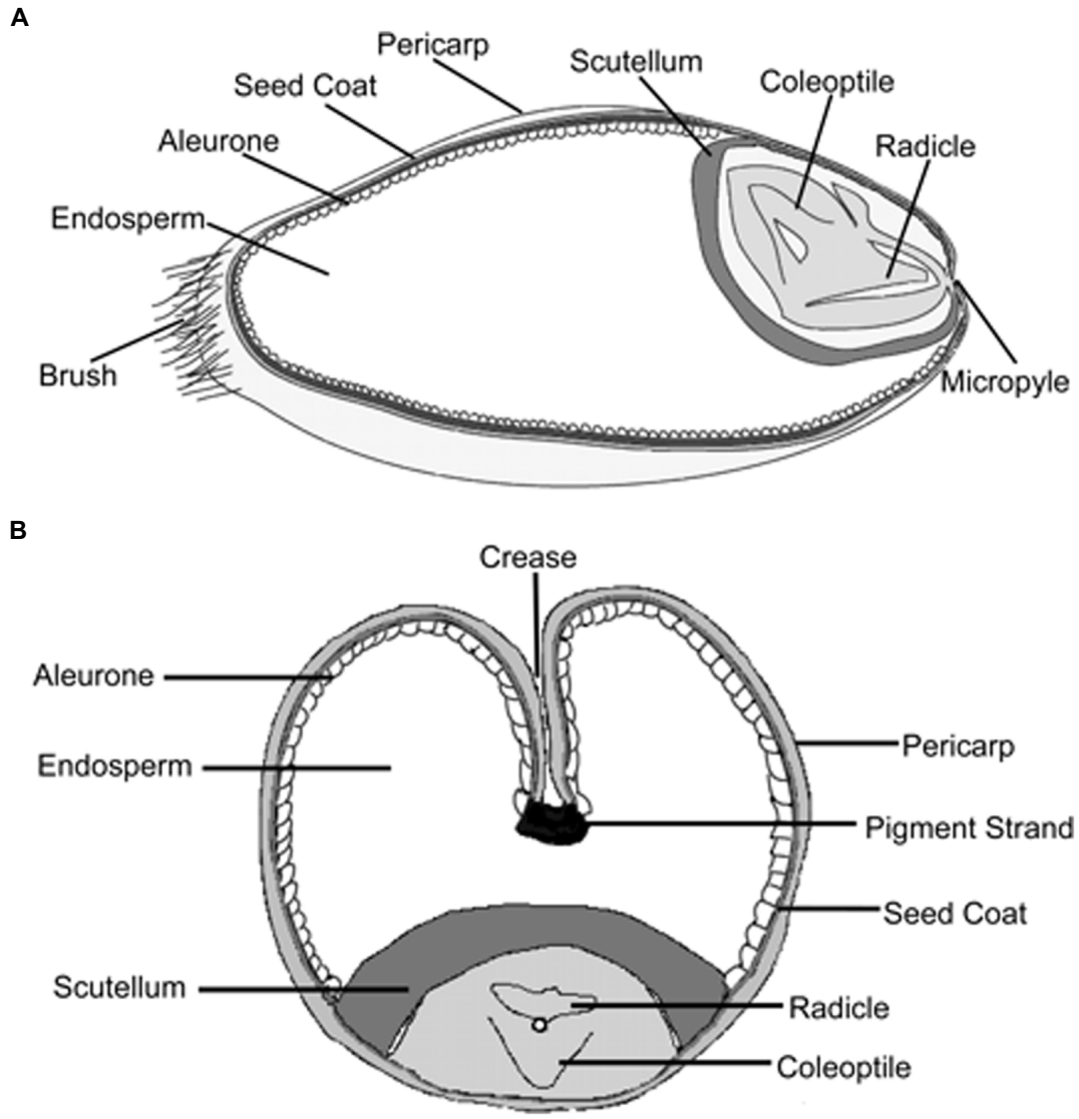
Wheat grain structure in longitudinal and transverse section.

Wheat fiber is made up of several layers of cells which, when the seed is dry, adhere so firmly to one another that they are removed by the milling process in comparatively large pieces. By shifting and other mechanical means, almost all the embryo and endosperm are removed. However, as the separation is never perfect, even the purest commercial bran always contains a little endosperm and possibly traces of embryo (67). Chemically, as well as structurally, bran differs significantly from the embryo and endosperm and, consequently, its nutritive value also differs. The longitudinal and transverse section of the wheat grain is shown in Figure 5 (68).

The bran, also known as the seed coat, which is present on the external layers of the wheat kernel and composed of numerous layers, is responsible for providing protection to the central part of the kernel. The seed coat is enriched with minerals and vitamin B (14). The bran is detached from the endosperm containing starch during the initial step of milling. The bran consists of fiber that is not soluble in water so as to protect the kernel and endosperm. It contains 53% of the cellulosic components.

Wheat fiber has a complicated chemical configuration; however, it comprises pentosans and cellulose, polymers founded on arabinose, and xylose firmly attached to the proteins. These elements are typical polymers found in the cell layers, like the aleuronic layer and the wheat cell wall. Carbohydrates and proteins both signify 16% of the bran’s entire dry mass. The value of minerals is somewhat high, at 72%.

The two outer strata of the grain, the pericarp and the seed cover, are composed of inactive hollow cells. The internal bran sheet, the aleuronic sheet, is packed with active contents of plant cells (69). This somewhat illustrates the elevated concentrations of carbohydrates and protein in the bran. Significant variation exists in the particular level of amino acids that is present in the flour and the aleurone layer. The level of proline and glutamine is nearly half, whereas arginine is triple, and histidine, asparagine, lysine, alanine, and glycine are double the level in flour (65).

The endosperm forms approximately 84% of the entire seed, its proportions varying with the plumpness of the grain. This part of the seed provides food for the growing embryo. Unlike the constituents of the embryo, those of the endosperm are relatively stable substances, designed by nature to remain unchanged until the germinating embryo draws on them for its first supply of food (21).

The endosperm is enclosed *via* the fused seed coat and the pericarp. In the external endosperm, the aleuronic sheet holds a unique configuration (70). It is made up of a single sheet of cubical cells. Proteins and enzymes are abundant in the aleuronic sheet and perform a critical role during propagation. The internal endosperm lacking an aleuronic sheet is observed to be the mealy endosperm. In contrast to the other parts of the seed, the endosperm is characterized by its very high starch content, which, together with the protein, equals nearly 89% of its whole composition (71). The endosperm largely encloses food assets required for the development of seedlings and is full of starch. Besides carbohydrates, the starchy endosperm consists of 15% fats and 13% proteins, such as globulins, albumins, glutenins, and gliadins. The amount of nutritional fibers and minerals is low, at 0 or 5% and 1 or 5%, respectively (66).

The germ on one side of the kernel is enriched with 25% proteins and 8–13% lipids. The level of minerals is relatively high (4–5%). Wheat germ is obtained as a by product during wheat milling (65).

### 1.7. Chemical constituents of *Triticum aestivum*

The main chemical components of wheat are given below.

#### 1.7.1. Vitamins and minerals

Vitamin B5, B1, B6, B3, B8, B2, B12, K, E, and A; ascorbic acid; boran; dry ascorbic acid; iodine; sodium; carotene; group 2 metals of the periodic table; magnesium; molybdenum; potassium; zinc; aluminium; copper; phosphorus; sulfur; Iron; and selenium (72).

#### 1.7.2. Enzymes

Superoxide dismutase, protease, amylase, lipase, transhydrogenase, cytochrome oxidase (73).

#### 1.7.3. Supplementary constituents

Amino acids, e.g., valine, asparagine, aspartic acid, alanine, glutamine, proline, methionine, glycine, phenylalanine, threonine, leucine, arginine, tryptophan, isoleucine, tyrosine, serine, histidine, and lysine; mucopolysaccharides; chlorophyll; P4D1, i.e., glucoprotein; bioflavonoids, such as apigenin, luteolin, and quercitin; laetrile; indole complexes; and choline, i.e., amygdalin (74–76).

### 1.8. Nutritional attributes of wheat

Wheat grains and their products are significant constituents of our daily diet. The average wheat consumption is 318 grams per person each day, making up 83% of the overall cereal consumption (72).

Wheat contributes a larger percentage of protein than energy to the nutritional requirement of an adult male. Alone, it can fulfill the daily requirement of niacin and thiamine. The majority of the daily riboflavin and iron requirement is fulfilled by the quantity of wheat recommended for an adult male (Table 3).

**Table 3:** Nutrients and calories supplied by the wheat as % of suggested daily allowance for adult male (77).

	Calories(kcal)	Thiamin	Protein	Niacin	Riboflavin	Iron
% of RDA	42	168	79	95	54	56

Wheat is predominantly considered a source of protein, vitamins, calories, and minerals. It is comparable with various cereals in nutritional content. Its protein content is higher than sorghum, rice, and maize and about equivalent to that of other cereals. The protein content is influenced by a variety of cultural and environmental conditions, such as soil temperature, moisture, availability of nitrogen, and method of cultivation. The percentage of protein in wheat can be influenced to a certain extent by the time of fertilizer application and fertilizer type (72).

The nutritional content of protein is estimated not simply based on the concentration of protein but also the amino acid equilibrium within the protein. During human digestion, protein is broken down into its constituents, absorbed by the bloodstream, and then assembled again to form different types of protein required by the body for growth, maintenance, and repair (21). Eight amino acids are vital for humans as the body is unable to produce them and must take them from food.

The biological significance of wheat is determined by limiting essential amino acids. These amino acids become deficient due to the body’s increased requirements. Lysine is the deficient amino acid in wheat (64). During the process of milling, one-third of the total protein is removed along with lysine as the majority of the protein and lysine is present in the bran and the germ (14). There is an inverse relationship between the quantity of protein in grain and the quantity of lysine/grams of protein (77).

### 1.9. Wheat quality

Wheat quality has two main characteristics: external quality and internal quality. External quality involves freedom from foreign material and weather damage, type, and purity of color. These factors are used to separate wheat into visual grades (72). Internal factors involve parameters such as density, which is determined by evaluating the test weight; chemical composition, which includes protein content; moisture; and processing potential, which comprises milling quality, end-use quality, and enzyme activity (5).

#### 1.9.1. Classification and function of wheat proteins

Protein is regarded as the most significant nutrient for animals and humans, as the name of its origin indicates (“proteios,” meaning primary in Greek). The protein content varies from 10–18% of the entire dry mass of the wheat grain (72). Proteins determine the capability of wheat flour, which can be dispensed into diverse foodstuffs. Wheat proteins have an important role in carbon dioxide retention, dough development, and baking quality due to their quantitative qualitative and quantitative attributes (78). Mature wheat grains contain 8–20% protein. The proteins in wheat display great intricacy and diverse collaboration with one another, rendering them hard to describe (79, 80).

**Figure 6:**
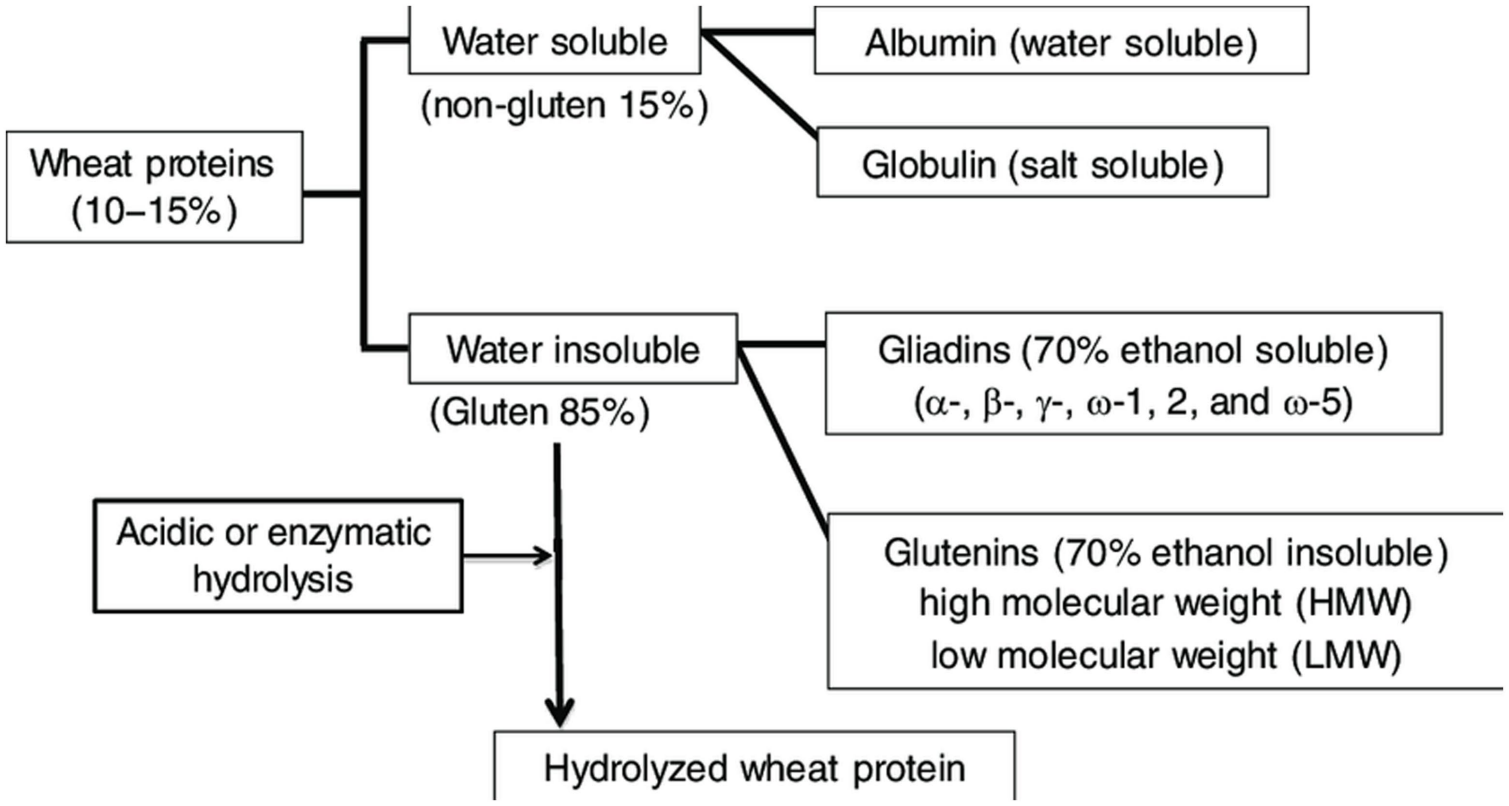
Types of wheat proteins.

Wheat proteins have been classified (Figure 6) (81) by their enforceability and solubility in various diluents. Cataloging was conducted on the basis of Thomas. D. Osborne’s work from the shift of the previous era (82). According to his method, serial withdrawal of crushed wheat kernels gives rise to protein properties as follows:

- Water soluble albumins
- Globulins, not soluble in natural water but soluble in diluted solution of sodium chloride while
- insoluble at high NaCl concentrations
- Gliadins, soluble in 70% ethanol
- Glutenins, soluble in diluted NaOH or acid solutions

Albumins and globulins are the smallest wheat proteins. The partition of globulins and albumins was not clear as originally recommended by Osborne. Gliadins and glutenins represent complex proteins of high molecular weight (83). The maximum number of wheat kernel proteins that are physiologically active has been found in globulin and albumin sets. In mueslis, they are stored in the germ, seed coverings, and aleuronic cells, with a low concentration in the starchy endosperm. Globulin and albumin constitute nearly 25% of all kernel proteins (79).

Traditionally, protein in wheat grains has been divided into prolamins and non-prolamins. The prolamins consist of gliadins and glutenins, while the non-prolamins include salt and water-soluble globulins. Albumin and globulin proteins concentrate during the initial phase of grain development, after which, the content of these proteins remains constant from 10–15 days after flowering (DAF) onwards, the albumins and globulins tend to accumulate in the emerging starchy endosperm from 10 to 15 DAF, involving primarily trypsin inhibitors, α, β-amylase, and triticins. The characteristics of wheat flour quality depend on the prolamin content and composition in the endosperm, whereas the role of albumins and globulins in the development of flour quality is not defined as well as that of prolamins (66).

Albumins and globulins are primarily metabolic enzymes, which have a role in numerous metabolic events during the course of grain filling, comprising starch synthesis, protein synthesis, folding, and energy metabolism. Storage proteins (gliadins and glutenins) constitute approximately 75% of the overall protein content. Wheat crops accumulate proteins in this way for seedling usage in advance. They are usually found in the starchy endosperm, not in the germ or seed coat sheet. Wheat storage proteins are technically dynamic. They lack enzyme action, but they perform a role in dough development; for example, these proteins are able to hold gas, generating soft baked foodstuff (66).

Albumins and the wheat endosperm’s globulin cover 20–25% of the total grain protein. Globulins and albumins have an excellent amino acid equilibrium with regard to nutrition. Several of these such proteins (enzymes) are involved in metabolic actions (79).

Wheat is exclusive among the palatable kernels, as its flour possesses a complex protein known as gluten, which, when prepared as dough, has viscous and elastic characteristics and is essential for manufacturing leavened bread. The rheological characteristics of gluten are required not merely for bread manufacture but for a broad range of foodstuff that is only prepared using wheat, like cookies, pastries, pitta bread, pasta, noodles, etc. The proteins in gluten include monomeric and polymeric gliadins and glutenins. Glutenins and gliadins are considered the main storing proteins of wheat, representing around 75–85% of the overall seed proteins, with a ratio of nearly 1:1 in bread and common wheat. They are enriched with proline, glutamine, asparagine, and arginine, but nutritionally significant amino acids, like tryptophan, lysine, and methionine, are present in small amounts (84).

The gliadins, which represent 30–40% of all total proteins in flour, are a polymorphic blend of proteins that are soluble in 70% alcohol. They are separated into alpha, beta, gamma, and omega gliadins, with an MW of 30–80 kilo Daltons, as defined by sodium dodecyl sulfate-polyacrylamide gel electrophoresis. The omega gliadin MW is in the range of 46–74 kilo Daltons, while alpha, beta, and gamma are low molecular weight gliadins, ranging from 30–45 kDa by amino acid sequencing and SDS-PAGE. Recent methodology has revealed a close link between alpha and beta gliadins and, thus, these are frequently called alpha-type gliadins (82).

Gliadins are freely soluble in dilute alcohols, except glutenin polymers; however, their subunits have the ability to be dissolved in a similar way to the gliadins. The subunits of glutenin can be acquired through the treatment of glutenin using a disulfide reducing agent; for example, β-mercapto-ethanol or dithiothreitol. Gliadins and glutenin subunits both have unexpectedly high levels of glutamine and proline. Hence, it has been suggested that these storing proteins be called ‘prolamins’, as they display strong similarities with most storage proteins in associated cereals, such as rye or barley. Residues of cysteine have an important role in the structure of gliadins and glutenin subunits. These residues have a role in either disulfide bonds inside similar or different polypeptides, i.e., intra-chain disulfide bonds, or inter-chain disulfide bonds (85).

Gliadins have shown an extremely varied fusion of a monomeric form of gluten proteins. Three anatomically different gliadins, alpha, gamma, and omega, can be illustrated. The evaluation of amino acid sequences has shown that α-and γ-gliadins are linked to low-molecular-weight glutenin subunits. For that reason, they have been categorized as ‘prolamins enriched with sulfur’. Residues of cysteines are situated at alpha type six remains of cysteine, and gamma type eight remains of cysteine gliadins have been found at extremely preserved sites and have a role in preserved intra-chain bonds of disulfide. Conversely, cysteine residues are absent in ω-type gliadins and possess a very small amount of methionine. So, these gliadins have been termed ‘sulfur-poor prolamins’ (73).

Polymers of glutenin are composed only of polypeptides related through the disulfide bonds present between the molecules, which account for approximately 45% of the total protein inside the kernel endosperm. Wheat protein consists of two types of subunits, the LMW 10,000–70,000 Da and the HMW subunits of glutenin 80,000 to 130,000 Da. Studies on glutenin genetics of wheat have shown the presence of high-molecular-weight glutenin subunit genes on the 1A, 1B, and 1D (extended chromosome arm) at the Glu-B1, Glu-D1, and Glu-A1 positions, respectively. Tightly linked genes (two) are found in each Glu-1 locus encoding x or y subunit types. In *Triticum aestivum*, the Glu-A1 locus encodes null (N) subunit and 1Ax, while the Glu-B1 locus commonly codes for 1Bx and 1By. Occasionally, Glu-B1 codes for 1Bx or 1By subunits, whereas the Glu-D1 locus codes for 1Dx and 1Dy subunits (85). As a result, for hexaploid wheat, three to five HMW-GS are usually produced by each genotype (85).

Electrophoretic studies have shown a significant alteration in mobility and the number of HMW-G subunits in pasta and bread wheat. LMW-GS constitutes around one-third of all the protein in the seeds and 60% of all the gluten protein (86). The LMW-S looks like gamma gliadins in sequence and consists of roughly 20–30% of the total protein, whereas the high-molecular-weight subunits constitute around 5–10% of the total protein (85).

Low molecular weight proteins that are abundant in cysteine might affect the viscoelastic characteristics of dough through disulfide or sulfhydryl exchange reactions with the proteins of gluten. Proteins capable of binding lipids can influence gluten-lipid protein interactions, and consequently, the functionality of protein in gluten (87). According to the evidence, stowage globulins that are polymeric in nature are related to few bread-making functions. In contrast to non-gluten proteins, gluten proteins are sparingly soluble in water or dilute salt solutions (85). A low quantity of ionizable side chain amino acids and an elevated level of non-polar amino acids and glutamine are the factors that contribute to its low solubility. The latter has better hydrogen bonding capabilities (73).

#### 1.9.2. Gluten proteins and wheat flour’s bread-making function

Proteins of gluten principally define the bread-making capability of wheat flour. A gluten protein allows the formation of cohesive viscoelastic dough when the flour is mixed with water and is able to retain gas produced in the process of fermentation or baking, forming bread’s exposed form configuration after baking. The viscoelastic attributes of dough that are crucial for bread manufacture are mainly regulated by the gluten proteins of wheat, but the collaboration between the gluten protein medium and the additional constituents of flour, such as lipids in flour (88), arabinoxylans (89), and non-gluten proteins, also have an influence on its viscoelastic properties. These properties of wheat gluten are altered further by the addition of oxidants, proteases, and reducing agents, which immediately modifies the gluten proteins, or by the addition of emulsifiers, lipids, and hemicelluloses, which alters gluten protein interactions.

The bread manufacturing ability of wheat flour is directly associated with the protein content in flour (90) and, therefore, with the gluten protein content, as this type of protein rises more than non-gluten protein due to its increased grain protein content. Therefore, an increased ‘amount’ of proteins in gluten is crucial. Nevertheless, the direct correlation between breadmaking performance and protein content relies on the genotype of the wheat, indicating that the quality of ‘gluten’ protein is also of significance in the overall quality of the wheat (82, 85).

A sufficient viscoelasticity equilibrium or the potential thereof is mandatory for excellent breadmaking. Inadequately flexible gluten will lead to decreased loaf size. Improved elasticity indicates increased loaf volume; however, excessively elastic gluten inhibits gas cell expansion (91), also causing decreased loaf size. Glutenin polymers are responsible for the strength and elasticity of dough (85). The glutenin elasticity is thought to be influenced by flexible stretching of actively and more favorably folded conformation of glutenin. Belton (92) suggested that gluten elasticity is due to non- covalent interaction mediate gluten elasticity primarily hydrogen bonds, inside and between single glutenin chains. Conversely, gliadins are plasticizers that deteriorate glutenin chain interaction (93), thus increasing the viscosity of dough. Therefore, the proportion of monomeric gliadin-polymeric glutenin defines the equilibrium in the viscoelasticity of dough and consequently influences the gluten protein value. Today, it is usually thought that quality differences are strongly influenced by alterations in the quality of glutenin (85).

### 1.10. Current challenges in wheat breeding programs and improvement approaches

An increase in the world’s population of almost 10 billion is expected by 2050, which will result in an increase in wheat demand at a rate of approximately 1.7 annually (94). Therefore, creating a sufficient supply response will persist as a policy challenge throughout the 21st century. Wheat breeders’ responsibility and their role in developing better varieties of wheat are becoming more significant in the improvement of crop production (95). Wheat productivity is vulnerable to newly developed diseases and pests, inadequate water resources, limited arable land, and quickly altering climatic situations (94, 96).

Numerous diseases, like rusts (stripe, leaf and stem rust, powdery mildew, spot blotch, Karnal bunt, and *Fusarium* head blight) severely hamper wheat productivity (97). Several researchers have described rusts as a major biotic stress in wheat, causing a wheat loss of 10–100%, depending upon virulence factor, resistant/susceptible cultivar genotype, initial time of infection, environment, pathogenesis ratio, and disease duration (98).

*Puccinia graminis* f. sp. *tritici* (stem rust) can result in a yield loss of up to 100%, and the sudden rise and spread of stem rust in Africa, known as *Ug99,* to Iran, the Middle East, and other countries is a severe concern for wheat production worldwide. *Tilletia indica* (Karnal bunt) disease is not only responsible for yield loss but also affects the quality of grain due to the infection of kernels. This disease was detected in various other countries, such as Mexico, Pakistan, India, Iran, Nepal, Afghanistan, Iraq, South Africa, and the United States (94).

*The Fusarium culmorum and Fusarium graminearum* species of *Fusarium* Head Blight/head scab cause grain to become infected with mycotoxins, such as nivalenol (NIV), deoxynivalenol (DON), and zearalenone (ZON). Yield losses occur due to shriveled grain, low test weight, and failure of seed formation (99). Spot blotch (SB), a vicious wheat leaf disease initiated by *Cochliobolus sativus* can cause a 70% yield loss. Composite quantitative inheritance of SB resistance has reduced breeding progress (100). Another harmful disease is the biotrophic fungus *Blumeria graminis*, a powdery mildew (PM), which is a universally occurring wheat foliar disease responsible for terrible yield loss (101).

In contrast to biotic stress resistance, the resistance gene plays a small role in defending wheat against insects due to the substantial effect of temperature and light on the existence and performance of the insects (94). Continuous damage to crop production caused by pests and diseases is one of the key limitations in wheat breeding and, therefore, food sufficiency globally. Aphids, termites, wheat midges, wheat weevil armyworms, Hessian flies, and cereal cyst nematodes (CCN) are the main arthropods feeding on wheat among various pests (102), making it essential to recognize novel genes and know their interactions and functions in resistance to CCN (103).

Abiotic stresses like drought, salt, and terminal heat stress are important as they limit wheat production and pose a substantial challenge to wheat breeding programs internationally (104). Climate change is another factor that has resulted in a wheat loss of 33% globally because of temperature increases and water shortages in wheat-growing areas (105, 106). The induction of chromosome restitution in meiosis at the time of male gamete development is a major problem caused by climate variation. Heat stress at the terminal stage of the wheat crop halts plant growth and the accumulation of starch, causing yield fickleness (94). On the other hand, global warming brutally disturbs weather patterns, ensuing temperature extremes, frequent frost, drought, and snowfall (94). Complex interactions of cellular and molecular mechanisms with whole-plant adaptation have limited breeding approaches to heat resistance (104).

The complex wheat genome and barriers in hybridization pose major challenges in identifying and understanding various gene functions, thus making the manipulation and characterization of traits of concern very difficult in the development of better varieties (107). Therefore, in order to understand several networks of genes and their jobs in the wheat genome, the continued characterization of traits of landraces and wild relatives is still needed for rapid progress in the improved development of cultivars (98).

Plant breeders need to find new resistance genes by manipulating wheat germplasm, which is essential in combatting such insect/pest diseases. Genome-wide association studies (GWAS) or QTL mapping can be employed to find genes with drought resistance in unexplored germplasm. The use of genes/transcription factors from wheat germplasm like DREB, NHX2, AVP1, and SHN1 and their associated markers is a viable method for producing salt-tolerant wheat genotypes (104). QTLs in combination with R-specific resistant genes provide effective and durable resistance in different environments (108). Both approaches are suggested to deal with climate change.

Precise pre-breeding and selection approaches need to be carefully designed and followed for the identification and exploitation of the most effective and resilient loci, pyramiding, and partially tolerant gene accumulation. Identification, cloning and modification, and transfer of various R genes to diverse crop species through conventional breeding methods, molecular Marker Assisted Selection (MAS), and biotechnological tools, such as OMICS (genomics, transcriptomics, proteomics, metabolomics, etc.), can be used to combat pests and diseases and achieve long-lasting resistance (108).

Tools in tissue culture techniques, like micropropagation, gametic embryogenesis (103, 109), somatic embryogenesis, cell suspension, and protoplast fusion facilitate the fast, large scale-cloning of high-value plants to produce pure lines (109). Apart from plant breeding technologies, some abiotic factors can be reversed through environmental management practices and developing microbe linkage to plants to biologically control pathogens (110, 111). Diethyl aminoethyl hexanoate application is found to increase plant tolerance to abiotic stress, such as cold (112) and salt stress, (113) whereas applying proline amino acid during plant adaptation increases tolerance to salinity stress (114).

The incorporation of *in vitro* approaches, like protoplast fusion (109), gametic embryogenesis (103), somatic embryogenesis, mutagenesis, and plant cell/tissue culture, and the current biotechnology practices, like synthetic biology, transgenic plants, gene editing (115), OMICS technologies, and interference RNAs (116, 117), can enhance tolerance to biotic and abiotic factors and control the depth of plant roots (103). Another way to increase resistance is by using microbial biotechnology to enhance plant nutrition and/or promote biocontrol against pathogens (110), applying diethyl aminoethyl hexanoate to achieve resistance to abiotic stress (116, 118), and using proline to increase salt tolerance (119) and improve poor water conditions (114).

Gene stacking can be used to combat disease-resistant genes and inherit them as a sole trait (120). The incorporation of even more disciplines is needed for breeding to reach the ultimate ‘deterministic’ phase and catch up with the situation in genomics, *in silico* breeding and phenomics, etc. Therefore, public sectors must incorporate novel technologies into Mendelian genetics and the principles of quantitative genetics in order to make dynamic alterations in crop production (94).

## Author contributions

AK: write-up and revision of manuscript. AH: planning, finalization of basic idea, and revision. MT: revision of manuscript. All authors contributed to the article and approved the submitted version.

## Conflict of interest

The authors declare that the research was conducted in the absence of any commercial or financial relationships that could be construed as a potential conflict of interest.

## Publisher’s note

All claims expressed in this article are solely those of the authors and do not necessarily represent those of their affiliated organizations, or those of the publisher, the editors and the reviewers. Any product that may be evaluated in this article, or claim that may be made by its manufacturer, is not guaranteed or endorsed by the publisher.

## References

[1] Kumar S Kumari P Kumar U Grover M Singh AK Singh R. Molecular approaches for designing heat tolerant Wheat. J Plant Biochem Biotechnol. (2013) 22:359–71. doi: 10.1007/s13562-013-0229-3

[2] Nawaz R Inamullah HA Ud Din S Iqbal MS Gürsoy S Kutbay HG. Agromorphological studies of local Wheat varieties for variability and their association with yield related traits. Pak J Bot. (2013) 45:1701–6.

[3] Kizilgeci F Yildirim M Islam MS Ratnasekera D Iqbal MA Sabagh AE. Normalized difference vegetation index and chlorophyll content for precision nitrogen Management in Durum wheat cultivars under semi-arid conditions. Sustainability. (2021) 13:3725. doi: 10.3390/su13073725

[4] Anam Khalid AH Shamim S Ahmad J. Divergence in single kernel characteristics and grain nutritional profiles of wheat genetic resource and association among traits. Front Nutr. (2022) 8:805446. doi: 10.3389/fnut.2021.805446 35223936 PMC8864306

[5] Khan MNN Ahmad Z Ghafoor A. Genetic diversity and disease response of rust in bread Wheat collected from Waziristan agency, Pakistan. Int J Biodivers Conserv. (2011) 3:10–8. doi: 10.5897/IJBC.9000067

[6] Riaz MW Yang L Yousaf MI Sami A Mei XD Shah L. Effects of heat stress on growth, physiology of plants, yield and grain quality of different spring wheat (*Triticum Aestivum* L.) genotypes. Sustainability. (2021) 13:2972. doi: 10.3390/su13052972

[7] Breiman A Graur D. Wheat evolution. Israel J Plant Sci. (1995) 43:85–98. doi: 10.1080/07929978.1995.10676595

[8] Ali A Khaliq T Ahmad A Ahmad S Malik A Rasul F. How Wheat responses to nitrogen in the field. Crop Environ. (2012) 3:71–6.

[9] Erenstein O Jaleta M Mottaleb KA Sonder K Donovan J Braun H-J. Global trends in Wheat production, consumption and trade In: Reynolds MP, Braun HJ, editors. Wheat Improvement. Midtown Manhattan, NY: Springer (2022). 47–66.

[10] Sharma S Shrivastav VK Shrivastav A Shrivastav B. Therapeutic potential of Wheat grass (*Triticum Aestivum* L.) for the Treatmentof chronic diseases. South Asian. J Exp Biol. (2013) 3:308–13. doi: 10.38150/sajeb.3(6).p308-313

[11] Garg M Sharma A Vats S Tiwari V Kumari A Mishra V. Vitamins in cereals: a critical review of content, health effects, processing losses, bioaccessibility, fortification, and biofortification strategies for their improvement. Front Nutr. (2021) 8:586815. doi: 10.3389/fnut.2021.586815 34222296 PMC8241910

[12] Ikhtiar K Alam Z. Nutritional composition of Pakistani wheat varieties. J Zhejiang Univ Sci B. (2007) 8:555–9. doi: 10.1631/jzus.2007.B0555, PMID: 17657856 PMC1934949

[13] Urade R Sato N Sugiyama M. Gliadins from wheat grain: an overview, from primary structure to nanostructures of aggregates. Biophys Rev. (2018) 10:435–43. doi: 10.1007/s12551-017-0367-2, PMID: 29204878 PMC5899726

[14] Siddiqi RA Singh TP Rani M Sogi DS Bhat MA. Diversity in grain, flour, amino acid composition, protein profiling, and proportion of Total flour proteins of different Wheat cultivars of North India. Front Nutr. (2020) 7:141. doi: 10.3389/fnut.2020.00141, PMID: 33015119 PMC7506077

[15] Hassan G Gul R. Diallel analysis of the inheritance pattern of agronomic traits of bread Wheat. Pak J Bot. (2006) 38:1169–75.

[16] Bibi K Inamullah HA Din S Muhammad F Iqbal MS. Characterization of Wheat genotypes using randomly amplified polymorphic DNA markers. Pak J Bot. (2012) 44:1509–12.

[17] Khalid A Hameed A Tahir MF. Estimation of genetic divergence in Wheat genotypes based on agro-morphological traits through agglomerative hierarchical clustering and principal component analysis. Cereal Res Commun. (2022) 50:1–8. doi: 10.1007/s42976-022-00287-w

[18] Dubcovsky J Dvorak J. Genome plasticity a key factor in the success of Polyploid Wheat under domestication. Science. (2007) 316:1862–6. doi: 10.1126/science.1143986, PMID: 17600208 PMC4737438

[19] Hawkesford MJ Araus JL Park R Calderini D Miralles D Shen T. Prospects of doubling global Wheat yields. Food and Energy Security. (2013) 2:34–48. doi: 10.1002/fes3.15

[20] Shewry P. Wheat. J Exp Botany. (2009) 60:1537–53. doi: 10.1093/jxb/erp058 19386614

[21] de Sousa T Ribeiro M Sabença C Igrejas G. The 10, 000-year success story of Wheat! Foods. (2021) 10:2124. doi: 10.3390/foods10092124 34574233 PMC8467621

[22] Pour-Aboughadareh A Kianersi F Poczai P Moradkhani H. Potential of wild relatives of Wheat: ideal genetic resources for future breeding programs. Agronomy. (2021) 11:1656. doi: 10.3390/agronomy11081656

[23] Nalam VJ Vales MI Watson CJ Kianian SF Riera-Lizarazu O. Map-based analysis of genes affecting the brittle rachis character in tetraploid wheat (*Triticum Turgidum* L.). Theor Appl Genet. (2006) 112:373–81. doi: 10.1007/s00122-005-0140-y, PMID: 16328232

[24] Simons KJ Fellers JP Trick HN Zhang Z Tai Y-S Gill BS. Molecular characterization of the major wheat domestication gene Q. Genetics. (2006) 172:547–55. doi: 10.1534/genetics.105.044727, PMID: 16172507 PMC1456182

[25] Abdulkadir AR. Dpph antioxidant activity, Total phenolic and Total flavonoid content of different part of Drumstic tree (Moringa Oleifera lam.). J Chem Pharm Res. (2015) 7:1423–8.

[26] Gohar S Sajjad M Zulfiqar S Liu J Wu J. Domestication of newly evolved Hexaploid Wheat—a journey of wild grass to cultivated Wheat. Front Genet. (2022) 13:2931. doi: 10.3389/fgene.2022.1022931, PMID: 36263418 PMC9574122

[27] Flavell R Bennett M Smith J Smith D. Genome size and the proportion of repeated nucleotide sequence DNA in plants. Biochem Genet. (1974) 12:257–69. doi: 10.1007/BF00485947, PMID: 4441361

[28] Pourkheirandish M Dai F Sakuma S Kanamori H Distelfeld A Willcox G. On the origin of the non-brittle rachis trait of domesticated einkorn Wheat. Front Plant Sci. (2018) 8:2031. doi: 10.3389/fpls.2017.02031, PMID: 29354137 PMC5758593

[29] Gupta P Mir R Mohan A Kumar J. Wheat genomics: present status and future prospects. Int J Plant Genom. (2008) 2008:1–36. doi: 10.1155/2008/896451 PMC2397558 18528518

[30] Xynias IN Mylonas I Korpetis EG Ninou E Tsaballa A Avdikos ID. Durum Wheat breeding in the Mediterranean region: current status and future prospects. Agronomy. (2020) 10:432. doi: 10.3390/agronomy10030432

[31] Kumar A Singh A Singh V Verma R Singh K. Influence of moisture regimes and fertility level on root and qualitative studies of Wheat (*Triticum Aestivum* L.) under late sown condition. Biol Forum. (2022) 14:1559–62.

[32] Haudry A Cenci A Ravel C Bataillon T Brunel D Poncet C. Grinding up Wheat: a massive loss of nucleotide diversity since domestication. Mol Biol Evol. (2007) 24:1506–17. doi: 10.1093/molbev/msm077, PMID: 17443011

[33] Wrigley C. Wheat: a unique grain for the world In: Khan K Shewry PR, editors. Wheat: Chemistry and Technology. St. Paul, MN: American Association of Cereal Chemists (2009). 1–17.

[34] Gooding M Khan K Shewry P. The Wheat crop. Wheat: Chemistry and Technology. (2009). 4th Edn: Amsterdam: Elsevier, 19–49.

[35] Edwards MA. (2010). *Morphological features of Wheat grain and genotype affecting flour yield*. PhD thesis. Lismore, NSW: Southern Cross University.

[36] Atwell W Finnie S. Wheat Flour. 2nd ed. Amsterdam: Elsevier (2016).

[37] Katyal M Singh N Virdi AS Kaur A Chopra N Ahlawat AK. Extraordinarily soft, medium-hard and hard Indian wheat varieties: composition, protein profile, dough and baking properties. Food Res Int. (2017) 100:306–17. doi: 10.1016/j.foodres.2017.08.050, PMID: 28888455

[38] Gadaleta A Giancaspro A Blechl AE Blanco A. A transgenic durum Wheat line that is free of marker genes and expresses 1dy10. J Cereal Sci. (2008) 48:439–45. doi: 10.1016/j.jcs.2007.11.005

[39] Gadaleta A Blechl A Nguyen S Cardone M Ventura M Quick J. Stably expressed D-genome-derived Hmw Glutenin subunit genes transformed into different durum Wheat genotypes change dough mixing properties. Mol Breed. (2008) 22:267–79. doi: 10.1007/s11032-008-9172-8

[40] Payne PI. Genetics of Wheat storage proteins and the effect of allelic variation on bread-making quality. Annu Rev Plant Physiol. (1987) 38:141–53. doi: 10.1146/annurev.pp.38.060187.001041

[41] Loukoianov A Yan L Blechl A Sanchez A Dubcovsky J. Regulation of Vrn-1 Vernalization genes in Normal and transgenic Polyploid Wheat. Plant Physiol. (2005) 138:2364–73. doi: 10.1104/pp.105.064287, PMID: 16055679 PMC1183422

[42] Reddy L Allan R Campbell K. Evaluation of cold hardiness in two sets of near isogenic lines of Wheat (*Triticum Aestivum*) with polymorphic vernalization alleles. Plant Breed. (2006) 125:448–56. doi: 10.1111/j.1439-0523.2006.01255.x

[43] Carson G Edwards N Khan K Shewry P. Criteria of Wheat and flour quality. Wheat: Chemistry and Technology (2009) 4th Edn: Amsterdam: Elsevier, 97–118.

[44] Evans LT Wardlaw IF Fischer RA. Wheat. In: Evans LT, editor. Crop Physiology. London, New York and Melbourne: Cambridge University Press (1975). p. 101–149.

[45] Feng T Xi Y Zhu Y-H Chai N Zhang X-T Jin Y. Reduced vegetative growth increases grain yield in spring Wheat genotypes in the dryland farming region of north-West China. Agronomy. (2021) 11:663. doi: 10.3390/agronomy11040663

[46] Kirby EM Appleyard M. Cereal Development Guide. 2nd ed. Stoneleigh, Warwickshire: Arable Unit, National Agricultural Centre (1984).

[47] Hadjichristodoulou A Della A Photiades J. Effect of sowing depth on plant establishment, Tillering capacity and other agronomic characters of cereals. J Agric Sci. (1977) 89:161–7. doi: 10.1017/S0021859600027337

[48] Kronenberg L Yu K Walter A Hund A. Monitoring the dynamics of Wheat stem elongation: genotypes differ at critical stages. Euphytica. (2017) 213:1–13. doi: 10.1007/s10681-017-1940-2

[49] Patrick J. Vascular system of the stem of the Wheat plant II development. Australian J Botany. (1972) 20:65–78.

[50] Johnson JW Austin KL Jones GS Davis GH King TM. Efficacy of 17α-Hydroxyprogesterone Caproate in the prevention of premature labor. N Engl J Med. (1975) 293:675–80. doi: 10.1056/NEJM197510022931401 1099445

[51] Shang Q Wang Y Tang H Sui N Zhang X Wang F. Genetic, hormonal, and environmental control of Tillering in Wheat. Crop J. (2021) 9:986–91. doi: 10.1016/j.cj.2021.03.002

[52] Russell K Van Sanford DA. Breeding Wheat for resilience to increasing nighttime temperatures. Agronomy. (2020) 10:531. doi: 10.3390/agronomy10040531

[53] Poehlman JM. Breeding Field Crops. New York: Henry Holt and Company Inc (1959). 427 p.

[54] Mandy G. Pflanzenzuechtung, Kurz Und Bundig. Budapest: Deutscher Landwirtschaftsverlag (1970).

[55] Kirby E Appleyard M. Development of the Cereal Plant. The Yield of Cereals Royal Agriculture Society of England, London (1983): 1–3.

[56] Kirby E. Ear development in spring Wheat. J Agric Sci. (1974) 82:437–47. doi: 10.1017/S0021859600051339

[57] De Vries AP. Flowering biology of Wheat, particularly in view of hybrid seed production—a review. Euphytica. (1971) 20:152–70. doi: 10.1007/BF00056076

[58] Brocklehurst P Moss J Williams W. Effects of irradiance and water supply on grain development in Wheat. Ann Appl Biol. (1978) 90:265–76. doi: 10.1111/j.1744-7348.1978.tb02635.x

[59] Impa SM Vennapusa AR Bheemanahalli R Sabela D Boyle D Walia H. High night temperature induced changes in grain starch metabolism alters starch, protein, and lipid accumulation in winter Wheat. Plant Cell Environ. (2020) 43:431–47. doi: 10.1111/pce.13671, PMID: 31702834

[60] Simmons SR. Growth, development, and physiology In: Heyne EG, editor. Wheat and Wheat Improvement. Madison, WI: American Society of Agronomy, Inc (1987). 77–113.

[61] Huang J Wang Z Fan L Ma S. A Review of Wheat Starch Analyses: Methods, Techniques, Structure and Function. Int J Biol Macromol. (2022) 203:149. doi: 10.1016/j.ijbiomac.2022.01.149 35093434

[62] Ferrante A Savin R Slafer GA. Floret development and grain setting differences between modern durum wheats under contrasting nitrogen availability. J Exp Bot. (2013) 64:169–84. doi: 10.1093/jxb/ers320, PMID: 23162124 PMC3528029

[63] Austin R Jones H. The Physiology of Wheat, Part III. Cambridge, UK: Plant Breeding Institute (1975).

[64] Poutanen KS Kårlund AO Gómez-Gallego C Johansson DP Scheers NM Marklinder IM. Grains–a major source of sustainable protein for health. Nutr Rev. (2022) 80:1648–63. doi: 10.1093/nutrit/nuab084, PMID: 34741520 PMC9086769

[65] Sramkova Z Gregova E Sturdík E. Chemical composition and nutritional quality of Wheat grain. Acta Chimica Slovaca. (2009) 2:115–38.

[66] Belderok B Mesdag J Donner DA. Bread-Making Quality of Wheat: A Century of Breeding in Europe. Berlin: Springer Science & Business Media (2000).

[67] Onipe OO Jideani AI Beswa D. Composition and functionality of Wheat bran and its application in some cereal food products. Int J Food Sci Technol. (2015) 50:2509–18. doi: 10.1111/ijfs.12935

[68] Rathjen JR Strounina EV Mares DJ. Water movement into dormant and non-dormant Wheat (*Triticum Aestivum* L.) grains. J Exp Bot. (2009) 60:1619–31. doi: 10.1093/jxb/erp037, PMID: 19386615 PMC2671619

[69] Beres BL Rahmani E Clarke JM Grassini P Pozniak CJ Geddes CM. A systematic review of durum Wheat: enhancing production systems by exploring genotype, environment, and management (G× E× M) synergies. Front Plant Sci. (1665) 2020:568657. doi: 10.3389/fpls.2020.568657 PMC7658099 33193496

[70] Bao J Malunga LN. Compositional diversity in cereals in relation to their nutritional quality and health benefits. Front Nutr. (2021):8. doi: 10.3389/fnut.2021.819923 PMC8777119 35071305

[71] Osborne TB Mendel LB. The nutritive value of the Wheat kernel and its milling products. J Biol Chem. (1919) 37:557–601. doi: 10.1016/S0021-9258(18)87394-X

[72] Iqbal MJ Shams N Fatima K. Nutritional quality of wheat In: Ansari MR, editor. Wheat. London: Intech Open (2022)

[73] Wieser H Koehler P Scherf KA. The two faces of Wheat. Front Nutr. (2020) 7:517313. doi: 10.3389/fnut.2020.517313, PMID: 33195360 PMC7609444

[74] Kulkarni S Acharya R Nair A Rajurkar N Reddy A. Determination of elemental concentration profiles in tender wheatgrass (*Triticum Aestivum* L.) using instrumental neutron activation analysis. Food Chem. (2006) 95:699–707. doi: 10.1016/j.foodchem.2005.04.006

[75] Padalia S Drabu S Raheja I Gupta A Dhamija M. Multitude potential of wheatgrass juice (green blood): an overview. Chronicles Young Scientists. (2010) 1:23.

[76] Singh N Verma P Pandey B. Therapeutic potential of organic *Triticum Aestivum* Linn. (Wheat grass) in prevention and treatment of chronic diseases: an overview. Int J Pharm Sci Drug Res. (2012) 4:10–4.

[77] Khan M. Nutritional Attributes of Wheat. Birmingham, AL: Progressive Farming (1984).

[78] Irshad M Idrees M Saeed A Muhammad BR Ahmad S Naeem R. Physiochemical trace elements and protein profiling of different Wheat varieties of Pakistani origin. Golden Res Thoughts. (2013) 3:1–8.

[79] Zilic S Barac M Pesic M Dodig D Ignjatovic-Micic D. Characterization of proteins from grain of different bread and durum Wheat genotypes. Int J Mol Sci. (2011) 12:5878–94. doi: 10.3390/ijms12095878, PMID: 22016634 PMC3189758

[80] Khalid A Hameed A. Characterization of Pakistani Wheat germplasm for high and low molecular weight Glutenin subunits using Sds-Page. Cereal Res Commun. (2019) 1:1–11. doi: 10.1556/0806.47.2019.013

[81] Chinuki Y Morita E. Wheat-dependent exercise-induced anaphylaxis sensitized with hydrolyzed Wheat protein in soap. Allergol Int. (2012) 61:529–37. doi: 10.2332/allergolint.12-RAI-0494, PMID: 23093796

[82] Shewry P. What is gluten—why is it special? Front Nutr. (2019) 6:101. doi: 10.3389/fnut.2019.00101 31334243 PMC6625226

[83] Sharma A Garg S Sheikh I Vyas P Dhaliwal H. Effect of Wheat grain protein composition on end-use quality. J Food Sci Technol. (2020) 57:2771–85. doi: 10.1007/s13197-019-04222-6, PMID: 32624587 PMC7316921

[84] Rosa-Sibakov N Poutanen K Micard V. How does Wheat grain, bran and Aleurone structure impact their nutritional and technological properties? Trends Food Sci Technol. (2015) 41:118–34. doi: 10.1016/j.tifs.2014.10.003

[85] Veraverbeke WS Delcour JA. Wheat protein composition and properties of Wheat Glutenin in relation to Breadmaking functionality. Crit Rev Food Sci Nutr. (2002) 42:179–208. doi: 10.1080/10408690290825510, PMID: 12058979

[86] Sharma D Saharan V Joshi A Jain D. Biochemical characterization of bread Wheat (*Triticum Aestivum* L.) genotypes based on Sds-Page. Triticeae Genom Gene. (2015):6.

[87] Mac Ritchie F Du Cros D Wrigley C. (1990). Flour polypeptides related to Wheat quality. Adv Cereal Sci Technol, Pomeranz Y., St Paul, MN: American Association of Cereal Chemists, Inc.) 79–145.

[88] Scherf KA. Immunoreactive cereal proteins in Wheat allergy, non-celiac gluten/Wheat sensitivity (Ncgs) and celiac disease. Curr Opin Food Sci. (2019) 25:35–41. doi: 10.1016/j.cofs.2019.02.003

[89] Roels S. Factors Governing Wheat and Wheat Gluten Functionality in Breadmaking and Gluten/Starch Separation. Belgium: Dissertationes de Agricultura (1998).

[90] Hoseney RC. Principles of Cereal Science and Technology. St. Paul, MN: American Association of Cereal Chemists (AACC) (1994).

[91] Andersson AA Andersson R Piironen V Lampi A-M Nyström L Boros D. Contents of dietary fibre components and their relation to associated bioactive components in whole grain Wheat samples from the Healthgrain diversity screen. Food Chem. (2013) 136:1243–8. doi: 10.1016/j.foodchem.2012.09.074, PMID: 23194520

[92] Belton P. Mini review: on the elasticity of Wheat gluten. J Cereal Sci. (1999) 29:103–7. doi: 10.1006/jcrs.1998.0227

[93] Khatkar B Bell A Schofield J. The dynamic rheological properties of glutens and gluten sub-fractions from wheats of good and poor bread making quality. J Cereal Sci. (1995) 22:29–44. doi: 10.1016/S0733-5210(05)80005-0

[94] Kumar S Jacob SR Mir RR Vikas V Kulwal P Chandra T. Indian Wheat genomics initiative for harnessing the potential of Wheat germplasm resources for breeding disease-resistant, nutrient-dense, and climate-resilient cultivars. Front Genet. (2022) 13:834366. doi: 10.3389/fgene.2022.834366 35846116 PMC9277310

[95] Vitale J Adam B Vitale P. Economics of wheat breeding strategies: focusing on Oklahoma hard red winter Wheat. Agronomy. (2020) 10:238. doi: 10.3390/agronomy10020238

[96] Beres BL Hatfield JL Kirkegaard JA Eigenbrode SD Pan WL Lollato RP. Toward a better understanding of genotype× environment× management interactions—a global Wheat initiative agronomic research strategy. Front Plant Sci. (2020) 11:828. doi: 10.3389/fpls.2020.00828, PMID: 32612624 PMC7308648

[97] Roy C Juliana P Kabir MR Roy KK Gahtyari NC Marza F. New genotypes and genomic regions for resistance to wheat blast in south Asian germplasm. Plan Theory. (2021) 10:2693. doi: 10.3390/plants10122693, PMID: 34961165 PMC8708018

[98] Bhardwaj SC Singh GP Gangwar OP Prasad P Kumar S. Status of Wheat rust research and Progress in rust management-Indian context. Agronomy. (2019) 9:892. doi: 10.3390/agronomy9120892

[99] Brar GS Brûlé-Babel AL Ruan Y Henriquez MA Pozniak CJ Kutcher HR. Genetic factors affecting fusarium head blight resistance improvement from introgression of exotic Sumai 3 alleles (including Fhb1, Fhb2, and Fhb5) in hard red spring Wheat. BMC Plant Biol. (2019) 19:1–19. doi: 10.1186/s12870-019-1782-2 31053089 PMC6499950

[100] Ayana GT Ali S Sidhu JS Gonzalez Hernandez JL Turnipseed B Sehgal SK. Genome-wide association study for spot blotch resistance in hard winter Wheat. Front Plant Sci. (2018) 9:926. doi: 10.3389/fpls.2018.00926, PMID: 30034404 PMC6043670

[101] Mwale VM Tang X Chilembwe E. Molecular detection of disease resistance genes to powdery mildew (*Blumeria Graminis* F. Sp. Tritici) in Wheat (*Triticum Aestivum*) cultivars. Afr J Biotechnol. (2017) 16:22–31. doi: 10.5897/AJB2016.15720

[102] Smiley RW Dababat AA Iqbal S Jones MG Maafi ZT Peng D. Cereal cyst nematodes: a complex and destructive Group of Heterodera Species. Plant Dis. (2017) 101:1692–720. doi: 10.1094/PDIS-03-17-0355-FE, PMID: 30676930

[103] Marli GKM. Current challenges in plant breeding to achieve zero hunger and overcome biotic and abiotic stresses induced by the global climate changes: a review. J Plant Sci Phytopathol. (2021) 5:053–7. doi: 10.29328/journal.jpsp.1001060

[104] Choudhary A Kaur N Sharma A Kumar A. Evaluation and screening of elite Wheat germplasm for salinity stress at the seedling phase. Physiol Plant. (2021) 173:2207–15. doi: 10.1111/ppl.13571, PMID: 34549444

[105] Dormatey R Sun C Ali K Coulter JA Bi Z Bai J. Gene pyramiding for sustainable crop improvement against biotic and abiotic stresses. Agronomy. (2020) 10:1255. doi: 10.3390/agronomy10091255

[106] Malhi GS Kaur M Kaushik P. Impact of climate change on agriculture and its mitigation strategies: a review. Sustainability. (2021) 13:1318. doi: 10.3390/su13031318

[107] Bekeko Z Mulualem T. Genetics of plant–pathogen interactions and resistance. J Gene Environ Resour Conservat. (2016) 4:123–34.

[108] Kaur B Bhatia D Mavi G. Eighty years of gene-for-gene relationship and its applications in identification and utilization of R genes. J Genet. (2021) 100:1–17. 34282731

[109] Gniech Karasawa MM Embryogenesis Gametic, Somatic Embryogenesis, plant cell cultures, and protoplast fusion: Progress and opportunities in biofuel production. Nascimento DD do Pickering WA Plant-Based Genetic Tools for Biofuels Production, Netherlands: Bentham Science Publishers. (2017). 15.

[110] Yadav AN Kour D Kaur T Devi R Guleria G Rana KL. Microbial biotechnology for sustainable agriculture: current research and future challenges. New and Future Developments in Microbial Biotechnology and Bioengineering, Amsterdam: Elsevier. (2020): 331–344.

[111] Okungbowa FI Shittu HO Obiazikwor HO. Endophytic bacteria: hidden protective Associates of Plants against biotic and abiotic stresses. Notulae Scientia Biologicae. (2019) 11:167–74. doi: 10.15835/nsb11210423

[112] Lu J Guan P Gu J Yang X Wang F Qi M. Exogenous Da-6 improves the low night temperature tolerance of tomato through regulating Cytokinin. Front Plant Sci. (2021) 11:599111. doi: 10.3389/fpls.2020.599111, PMID: 33613581 PMC7889814

[113] Zhang C He P Li Y Li Y Yao H Duan J. Exogenous diethyl Aminoethyl Hexanoate, a plant growth regulator, highly improved the salinity tolerance of important medicinal plant Cassia Obtusifolia L. J Plant Growth Regul. (2016) 35:330–44. doi: 10.1007/s00344-015-9536-3

[114] Ghaffari H Tadayon MR Bahador M Razmjoo J. Investigation of the proline role in controlling traits related to sugar and root yield of sugar beet under water deficit conditions. Agric Water Manag. (2021) 243:106448. doi: 10.1016/j.agwat.2020.106448

[115] Steinwand MA Ronald PC. Crop biotechnology and the future of food. Nature Food. (2020) 1:273–83. doi: 10.1038/s43016-020-0072-3

[116] Liu S Geng S Li A Mao Y Mao L. Rnai Technology for Plant Protection and its Application in Wheat. aBIOTECH. (2021) 2:365–74. doi: 10.1007/s42994-021-00036-3 36304420 PMC9590511

[117] Dalakouras A Wassenegger M Dadami E Ganopoulos I Pappas ML Papadopoulou K. Genetically modified organism-free RNA interference: exogenous application of RNA molecules in plants. Plant Physiol. (2020) 182:38–50. doi: 10.1104/pp.19.00570, PMID: 31285292 PMC6945881

[118] Zhang J Li S Cai Q Wang Z Cao J Yu T. Exogenous diethyl Aminoethyl Hexanoate ameliorates low temperature stress by improving nitrogen metabolism in maize seedlings. PloS One. (2020) 15:e0232294. doi: 10.1371/journal.pone.0232294, PMID: 32353025 PMC7192554

[119] Naliwajski M Skłodowska M. The relationship between the antioxidant system and proline metabolism in the leaves of cucumber plants acclimated to salt stress. Cells. (2021) 10:609. doi: 10.3390/cells10030609, PMID: 33801884 PMC7998282

[120] Chang YN Zhu C Jiang J Zhang H Zhu JK Duan CG. Epigenetic regulation in plant abiotic stress responses. J Integr Plant Biol. (2020) 62:563–80. doi: 10.1111/jipb.12901 31872527

